# Impact of Extracellular Vesicle Isolation Methods on Downstream miRNA Analysis in Semen: A Comparative Study

**DOI:** 10.3390/ijms21175949

**Published:** 2020-08-19

**Authors:** Marina Mercadal, Carolina Herrero, Olga López-Rodrigo, Manel Castells, Alexandre de la Fuente, Francesc Vigués, Lluís Bassas, Sara Larriba

**Affiliations:** 1 Human Molecular Genetics Group-Bellvitge Biomedical Research Institute (IDIBELL), 08908 Hospitalet de Llobregat, Barcelona, Spain; mamerlli@msn.com; 2 Translational Medical Oncology Group (Oncomet), Health Research Institute of Santiago de Compostela (IDIS), University Hospital of Santiago de Compostela (SERGAS), 15706 Santiago de Compostela, Spain; carolina.herrero@rai.usc.es; 3 Laboratory of Seminology and Embryology, Andrology Service-Fundació Puigvert, 08025 Barcelona, Spain; olopez@fundacio-puigvert.es (O.L.-R.); lbassas@fundacio-puigvert.es (L.B.); 4 Urology Service, Bellvitge University Hospital-ICS, 08908 Hospitalet de Llobregat, Barcelona, Spain; mcastells@bellvitgehospital.cat (M.C.); fvigues@bellvitgehospital.cat (F.V.); 5 NASASBIOTECH S.L., Santiago de Compostela Hospital (CHUS), 15706 Santiago de Compostela, Spain; alexandre.fuente.gonzalez@nasasbiotech.com

**Keywords:** semen, extracellular vesicles, exosomes, microvesicles, extracellular vesicle isolation methods, extracellular vesicle miRNA analysis, biomarker, diagnosis, prostate cancer

## Abstract

Seminal plasma (SP) contains a unique concentration of miRNA, mostly contained in small extracellular vesicles (sEVs) such as exosomes, some of which could be clinically useful for diagnosis and/or prognosis of urogenital diseases such as prostate cancer (PCa). We optimized several exosome-EV isolation technologies for their use in semen, evaluating EV purifying effectiveness and impact on the downstream analysis of miRNAs against results from the standard ultracentrifugation (UC) method to implement the use of SP sEV_miRNAs as noninvasive biomarkers for PCa. Our results evidenced that commercial kits designed to isolate exosomes/EVs from blood or urine are mostly applicable to SP, but showed quantitative and qualitative variability between them. *ExoGAG 3500× g* and the *miRCURY Cell/Urine/CSF 1500*× *g* methods resulted as equivalent alternative procedures to *UC* for isolating exosomes/sEVs from semen for nanoparticle characteristics and quality of RNA contained in vesicles. Additionally, the expression profile of the altered semen sEV-miRNAs in PCa varies depending on the EV isolation method applied. This is possibly due to different extraction techniques yielding different proportions of sEV subtypes. This is evidence that the exosome-EV isolation method has a significant impact on the analysis of the miRNAs contained within, with important consequences for their use as clinical biomarkers. Therefore, miRNA analysis results for EVs cannot be directly extrapolated between different EV isolation methods until clear markers for delineation between microvesicles and exosomes are established. However, EV extraction methodology affects combined models (semen exosome miRNA signatures plus blood Prostate specific antigen (PSA) concentration for PCa diagnosis) less; specifically our previously described (miR-142-3p + miR-142-5p + miR-223-3p + PSA) model functions as molecular marker from EVs from any of the three isolation methods, potentially improving the efficiency of PSA PCa diagnosis.

## 1. Introduction

Healthy and pathological cells both release various types of membranous structures, called extracellular vesicles (EVs), into the biological fluids which include not only blood plasma and urine but also semen, amongst other fluids [1]. EVs typically range in size from 40 to 500 nm and are generally classified according to their cellular origin and biogenesis into apoptotic bodies, microvesicles and exosomes. Most EV research has been focused on exosomes, small cell-derived vesicles (40–150 nm) of endocytic origin. Microvesicles are larger EVs (100–800 nm) formed by budding from the plasma membrane whereas apoptotic bodies (>800 nm) are secreted by cells that have initiated programmed cell death. These nanovesicles, regardless of the subcellular origin, represent mechanisms of intercellular communication and paracrine signalling as they can transfer biological molecules from producer cells to acceptor cells and are involved in both healthy and pathological biological processes [2]. EVs contain specific molecular profiles representing the cell of origin, thus they are good indicators of the pathophysiological state of cells. As a result, several scientific applications for the clinics have been proposed for these vesicles: they could be used as cell biopsies in fluids for diagnostics, as novel target for therapeutic intervention, or as drug delivery vehicles for macromolecule transport [3].

Specifically in mammals, it has been calculated that each ejaculate contains trillions of small EVs (sEVs) that are morphologically and molecularly consistent with exosomes. These originate from multiple cellular sources in the male reproductive tract namely: prostate, epididymis, seminal vesicles and the testis [4,5]. EVs, and exosomes in particular, carry a multitude of molecules, such as proteins, lipids and nucleic acids (DNA, mRNA, miRNA) [6], that have been extensively studied for their potential use as non-invasive disease biomarkers. In particular, miRNAs are small noncoding RNA (19–22 nucleotides) that act as negative post-transcriptional regulators of gene expression. The tissue-specific expression profile and the high stability of these molecules within exosomes highlight the potential use of exosomal miRNAs in biofluids as biomarkers for diagnostic and/or prognostic purposes. Specifically, recent studies from our group provide evidence that semen exosomes can be useful as a source of miRNA biomarkers for the diagnosis of urogenital diseases such as male infertility [7] and prostate cancer [8].

Currently, the most extensively used method for isolating EVs, including exosomes, from biofluids for research purposes is ultracentrifugation, which can be technically challenging. The ultracentrifugation method requires specialized equipment that may not be available in clinical laboratories, therefore it is not an obvious choice for clinical work. For this reason, alternative methods based on precipitation reagents and/or solid filtration phase which do not require specialised equipment, have been recently developed for the isolation of exosomes and other EVs. We aim to optimize the protocol of sEV isolation procedures for semen samples and evaluate their effectiveness as well as the effect on downstream miRNA analysis for prostate cancer, as an example of a urogenital pathological condition. Additionally, results from several exosome-EV isolation commercial kits are compared with the use of a “gold-standard” ultracentrifugation-based method to implement the use of semen exosomal/sEV-miRNAs as a diagnostic method in the clinical laboratory.

## 2. Results

### 2.1. Characterization of Nanovesicles by Size and Concentration

In order to characterize the different exosome-EV isolation methods, we first performed an evaluation of sEVs by measuring size and concentration of the isolated particles from healthy control (HCt) semen samples (Table 1).

**Figure 1:**
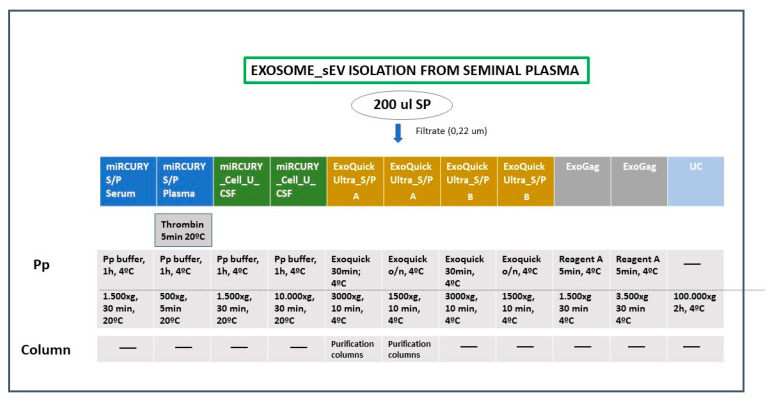
Technical conditions of the different exosome isolation methods applied. Semen-derived small extracellular vesicles (sEVs), including exosomes, were isolated using four precipitation-based reagents to compare with ultracentrifugation as a gold-standard technique. Different incubation and centrifugation conditions were tested. miRCURY S/P: *miRCURY Exosome Serum/Plasma* kit; miRCURY _Cell_U_CSF: *miRCURY Exosome Cell/Urine/CSF* kit; ExoQuick Ultra S/P: *ExoQuick Ultra EV Isolation* kit for Serum and Plasma; ExoGag: *ExoGAG* kit; UC: ultracentrifugation protocol; Pp: precipitation step.

The profiles of the particles isolated by four isolation methods with alternative precipitation conditions, compared to ultracentrifugation (*UC*) as the reference method (Figure 1), were evaluated.

**Figure 2:**
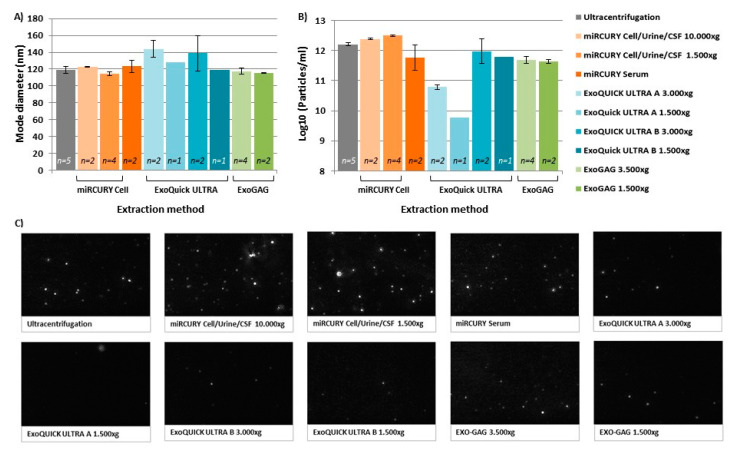
Nanoparticle tracking analysis (NTA) results of the EVs extracted from human semen using the different isolation methods and conditions. (**A**) Particle size is shown as the mode of EV diameter and (**B**) EV concentration is shown as the log10 of the number of particles/mL of the extracts obtained. Error bars represent the standard deviation (SD) of the mode/mean. (**C**) Representative NTA images of isolated vesicles. ExoQuick Ultra A refers to the protocol which includes precipitation and the purification columns whereas ExoQuick Ultra B only includes precipitation.

Nanoparticle tracking analysis (NTA) (Figure 2) provided evidence that isolated vesicles were within the expected size range for exosomes (50–250 nm), although an increased mean size and mode size was obtained for those extracted with *ExoQuick ULTRA (A* or *B* alternative protocols) kit, *miRCURY Cell/Urine/CSF 10,000× g* and *miRCURY Serum* (Supplementary Materials Table S1A,B) when compared with the *UC* method, so these methods resulted in a rather different distribution of undersized particles. *ExoGAG* and *miRCURY Cell/Urine/CSF 1500× g* exosome-EV isolation methods displayed very similar size profiles, including size mean and mode, to the ones obtained with the *UC* method (*p* > 0.05) (Supplementary Materials Table S1B, Figure 2A). In detail, ~90% of vesicles isolated by these three methods peaked below 250 nm, the average modal size (defined as the most frequently occurring EV size) being between 114–119 nm.

Additionally, NTA analysis revealed differences in the recovery rates for the methods used. Firstly, the *miRCURY S/P (protocol Plasma)* resulted in a low number of vesicles, below the recommended NTA detection levels (1 × 10^7^ particles/mL), making it the least efficient EV isolation method for SP. The *ExoQuick Ultra_S/P protocol A* (which includes the use of purification columns after the precipitation protocol) generated a low particle yield, both at 1500× *g* and 3000× *g*, isolating 266 and 25-fold fewer particles than the reference *UC* method, respectively, followed by the *miRCURY S/P* (*protocol Serum)* (Figure 2B) obtaining 2.8-fold fewer particles compared with *UC*. This suggests that the modifications made to the manufacturer’s indications have not been enough to provide a protocol with efficient EV isolation for SP. In contrast, the *miRCURY Cell/Urine/CSF* method, both at 10,000× *g* and at 1500× *g*, gave the best recovery rate for isolated vesicles from semen, resulting in 1.5–2-fold more particles respectively (Figure 2B), making it the most efficient EV isolation method.

**Figure 3:**
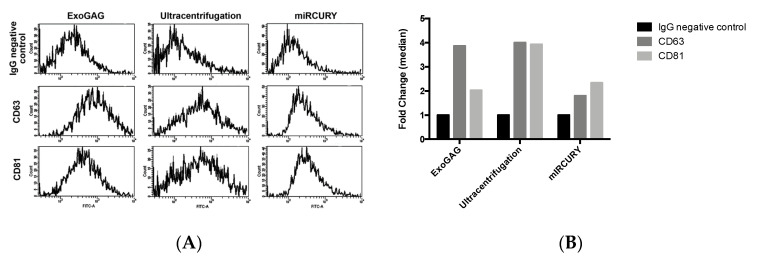
Characterization of sEVs by tetraspanin composition. sEVs isolated with ExoGAG (*ExoGAG 3500× g*), ultracentrifugation and miRCURY (*miRCURY Exosome Cell/Urine/CSF 1500× g*) protocols were tested. (**A**) Representative profiles of Alexa 488 fluorescence obtained by cytometry analysis. IgG (immunoglobulin G) was used as negative control of fluorescence. Positive CD63 and CD81 labelling was represented by a displacement of fluorescence peak curve. (**B**) Quantification of fluorescence represented by a fold-change of fluorescence intensity median with respect to IgG negative control. A specific CD63 and CD81 labelling of SP EVs isolated by ExoGAG is observed. A similar CD63 labelling was found when EVs were purified by ultracentrifugation, although a higher CD81 signal was obtained when compared with ExoGAG EVs. EVs isolated with miRCURY EV/exosome isolation kit showed a CD81 positive labelling.

Characterization of sEVs by their protein composition was additionally performed. Flow cytometry analysis of tetraspanins, as the most abundant proteins on EVs, showed positive labeling of CD63 and CD81 (Figure 3A) on vesicles isolated with either *UC*, *ExoGAG 3500× g*, *miRCURY Cell/Urine/CSF 1500× g* exosome-EV isolation methods (selected as they displayed very similar size profiles in the NTA analysis), although differences in the labelling signals are observed among them (Figure 3B).

Specifically, all samples were contrasted with antiIgG as unspecific marker (Figure 3A, upper panel). EVs isolated by *ExoGAG 3500× g* resulted in a positive CD63 and CD81 labelling represented by a displacement of fluorescence peak curve (Figure 3A, left panel). EVs purified by the *UC* technique showed a similar profile of CD63 and CD81 labelling with a displacement of fluorescence peak curve (Figure 3A, middle panel). However, EVs isolated by *miRCURY Cell/Urine/CSF 1500× g* exosome isolation kit only showed a clear positive CD81 labelling represented by a displacement of fluorescence peak curve (Figure 3A, right panel), and the CD63 fluorescence intensity was not enough to be considered as positive signal (CD63: IgG fold-change fluorescence intensity <2) (Figure 3B).

### 2.2. Nanovesicle RNA Quality and Quantity

Quality and quantity of RNA obtained from the nanoparticles was also assessed to evaluate the influence of each of the exosome-EV isolation methods on the RNA after EV recovery. Consistent with our results on EV characterization, RNA quality from the *ExoGAG 1500× g*; *ExoGAG 3500× g*; and *miRCURY Cell/Urine/CSF 1500× g* exosome-EV isolation methods displayed similar 260/280 ratios (1.76 ± 0.05; 1.79 ± 0.08 and 1.63 ± 0.11 respectively) to the *UC* technique (1.78 ± 0.17). Interestingly, the *miRCURY Cell/Urine/CSF 1500× g* exosome-EV isolation method gave a higher RNA concentration (Table 2), this is probably related to the higher vesicle yield obtained by this method (Figure 2); on the contrary the application of high speed (10,000× *g*) in the *miRCURY Cell/Urine/CSF* procedure resulted in a low concentration of RNA of bad quality.

Given the limited number of vesicles obtained by the *miRCURY S/P (protocol Plasma)*, we were unable to extract sufficient RNA for RNA analysis, and thus this method was definitively excluded from further analyses.

### 2.3. Pre-Study miRNA Expression Pattern

**Figure 4:**
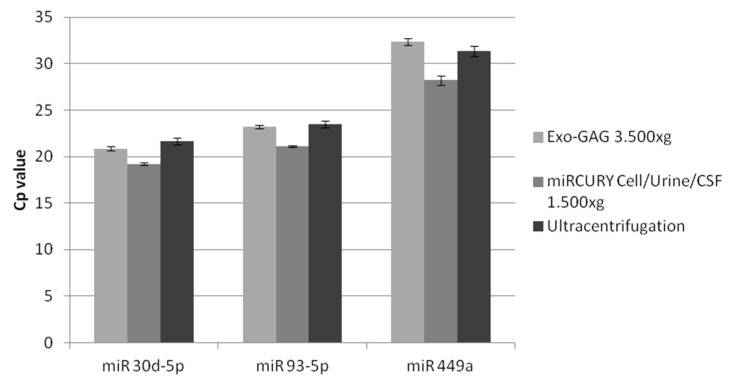
Semen exosome miR-30d-5p, miR-93-5p and miR-449a Cp values obtained by RT-qPCR. RNA was obtained from extracellular vesicle extracts isolated by using different techniques: *ExoGAG (3500× g)*, *miRCURY Cell/Urine/CSF (1500× g)* and *Ultracentrifugation (100,000× g)* methods. First-stranded cDNA specific for miRNA was obtained by RT of 50 µg in 10 µL using the Universal cDNA synthesis kit (Exiqon). The three semen exosomal miRNAs were amplified in a Lightcycler 96 instrument. No difference in the Cp values were found between the three methods. The values in the graph are the mean Cp values ± SD. RT-qPCR: quantitative reverse transcription PCR.

Next, we assessed whether SP sEV RNA of similar quality, which had been obtained from the different EV isolation methods, would result in similar miRNA quantitative real-time PCR (qPCR) amplification patterns from samples in physiological conditions. Accordingly, we subsequently analysed three miRNAs, which had previously been described as presenting quantifiable levels in semen exosomes (miR-30d-5p, miR-93-5p and miR-449a [7]), in samples from healthy control individuals. Although an equivalent amount of RNA from each of the EV isolation approaches was used, an increase of at least four raw Cp cycles was measured for the *miRCURY Cell/Urine/CSF at 10,000× g*, probably due to the poor quality of the RNA obtained with this approach. Slight differences in the Cp values were found among the rest of the different methods of exosome extraction (data not shown) and specifically among the most efficient exosome-EV extraction methods (Figure 4), that is *UC*, *ExoGAG 3500× g* and *miRCURY Cell/Urine/CSF 1500× g*.

Thus, according to the vesicle measurements, RNA assessment and miRNA quantification data, we chose the *ExoGAG 3500× g* and the *miRCURY Cell/Urine/CSF 1500× g* EV isolation methods for the next phase of the study, in which they were tested as equivalent alternative methods to *UC* for isolating exosomes/sEVs from semen.

### 2.4. Exosome/sEV-miRNA Expression Pattern in Prostate Cancer

In order to assess whether EV isolation methods influence the results of the miRNA analysis in semen for PCa, we analysed the expression of 16 human miRNAs (Supplementary Materials Table S2) in SP vesicles obtained by using the three exosome isolation methods mentioned above (*UC*, *miRCURY Cell/Urine/CSF 1500× g* and *ExoGAG 3500× g* methods). All of these miRNAs had been previously tested in semen exosomes isolated by *UC* [7,8] and some of them had been found to be differentially expressed in the prostates of PCa individuals (Supplementary Materials Table S2). MiRNA levels were assessed in semen vesicle RNA samples from 5 healthy control (HCt), 5 benign prostatic hyperplasia (BPH) and 9 prostate cancer (PCa) individuals (Table 1).

**Figure 5:**
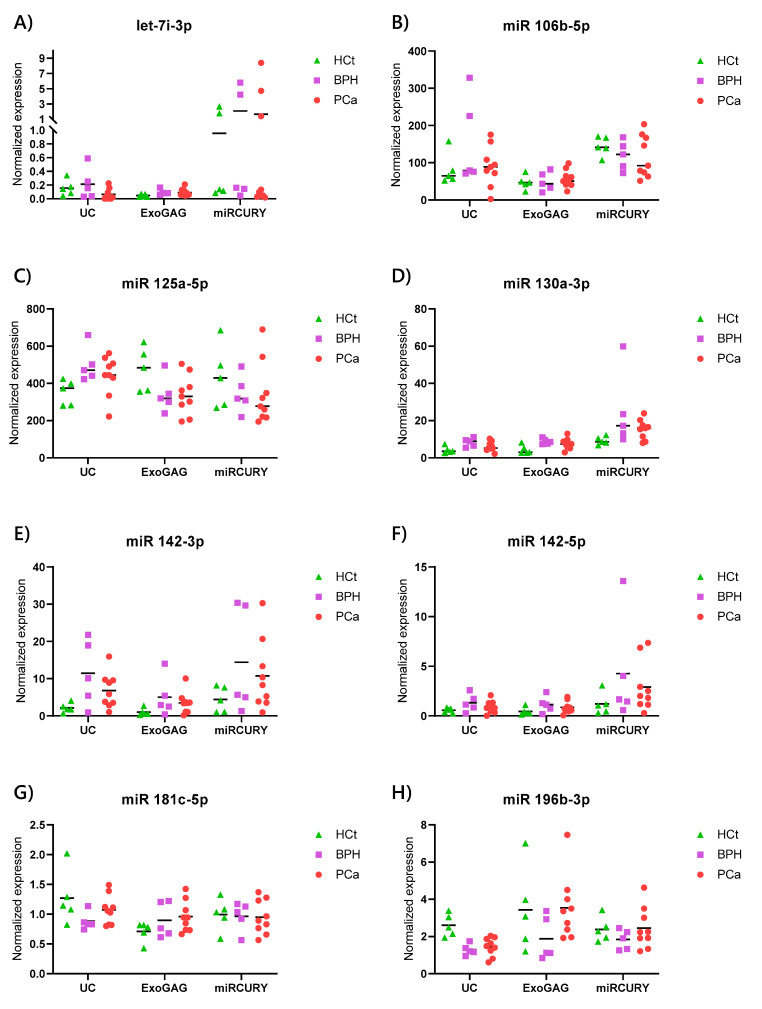

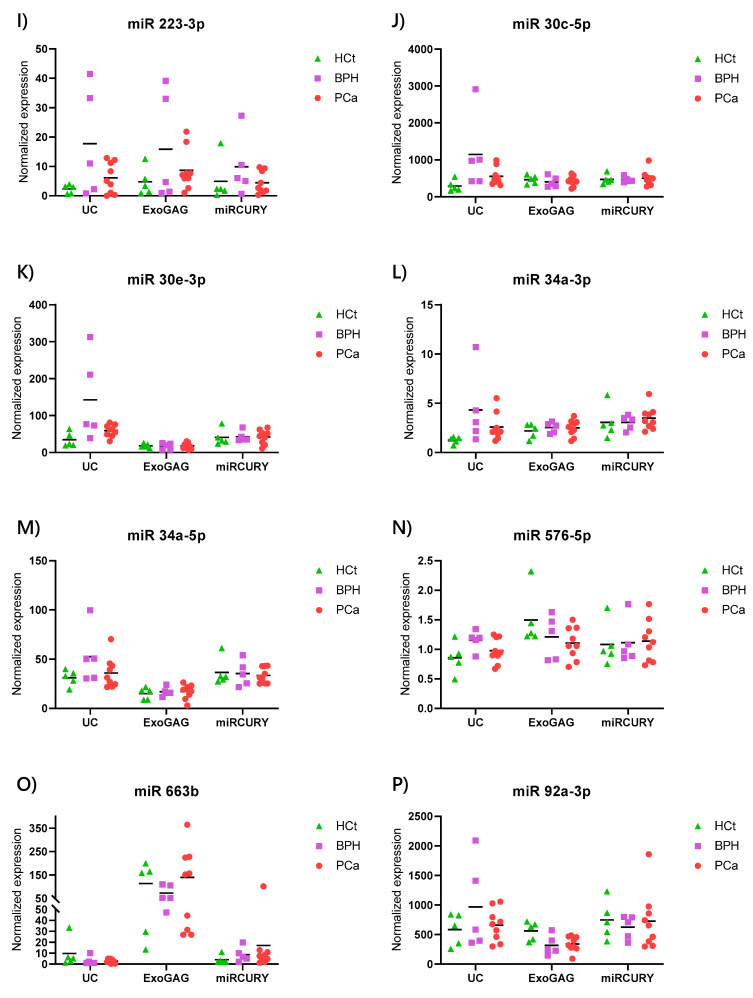
Expression profiling of the sixteen miRNAs (**A**, let7i-3p, **B**, miR-106b-5p, **C**, miR-125a-5p, **D**, miR-130a-3p, **E**, miR-142-3p, **F**, miR-142-5p, **G**, miR-181c-5p, **H**, miR-196b-3p, **I**, miR-223-3p, **J**, miR-30c-5p, **K**, miR-30e-3p, **L**, miR-34a-3p, **M**, miR-34a-5p, **N**, miR-576-5p, **O**, miR-663b, **P**, miR-92a-3p) in semen EV isolated by three methods: *UC* (ultracentrifugation), *ExoGAG* and miRCURY (*miRCURY Exosome Cell/Urine/CSF 1500× g)*. MiRNA expression levels were determined by RT-qPCR in healthy controls (HCt), benign prostate hyperplasia (BPH), and prostate cancer affected men (PCa). Data are shown as RQ values, which were calculated using the 2^dCp^ strategy and relative to the mean expression values of miR-576-5p and miR-181c-5p. The horizontal bar displays the median cellular expression level. RT-qPCR: quantitative reverse transcription PCR.

First, our results showed that miRNA levels showed a slight variation pattern in the different patient and control groups between the three methods. Specifically, the expression values for miR-142-3p, miR-196b-3p, miR-30c-5p and miR-34a-3p were statistically different between the PCa and HCt samples from vesicles obtained with the *UC* technique. None of the miRNAs presented differences in expression between the PCa and HCt groups from *miRCURY* purified EVs, whereas miR-142-3p and miR-92a-3p were found to be differentially expressed between PCa and HCt samples from *ExoGAG* extracted vesicles (Table 3; Figure 5). All of the differentially expressed miRNAs presented a fold-change increase or decrease of >1.5 between PCa and HCt samples. Thus, there is only one differentially expressed miRNA, miR-142-3p, between PCa and HCt EV samples for both the *UC* and *ExoGAG* methods.

Interestingly, we observe similar expression behaviour for some miRNAs tested when we used the different EV isolation methods. Some miRNAs, such as miR-142-3p, miR-142-5p and miR-223-3p showed the same trends in level of expression for all three methods (Figure 5), although the differences between PCa and HCt samples were not statistically significant for the three procedures. In contrast, some miRNAs were seen to be over-represented in vesicles obtained by one of the isolation methods. This is the case for miR-663b in vesicles extracted by *ExoGAG* (Figure 5), which indicates that the method has some kind of selectivity for vesicles containing this miRNA.

### 2.5. Diagnostic Performance of Combined sEV-miRNA-Based Diagnostic Classifiers for PCa

In our previous study [8], we showed semen exosome miRNA signatures that combined with blood Prostate specific antigen (PSA) concentration, can be used as molecular biomarkers with the potential to improve PCa diagnosis efficiency of PSA. The miRNAs included in the models (miR-142-3p, miR-142-5p and miR-223-3p) were obtained from sEVs isolated by the ultracentrifugation procedure. Thus, in the present study we further assessed whether these miRNA models were useful as biomarkers from sEVs obtained from the different EV isolation methods.

As mentioned above, miR-142-3p, miR-142-5p and miR-223-3p were over-expressed in PCa and BPH sEVs, showing the same trends in the level of expression for all three methods (Figure 5), although the differences between PCa and HCt samples were not statistically significant for the three procedures. Accordingly, the predictive accuracy of the miRNA multiplex model (miR-142-3p + miR-142-5p) was not statistically significant to discriminate PCa from non-PCa samples (Table 4), and the (miR-142-3p + miR-142-5p + miR-223-3p) model only showed diagnostic efficiency for the miRCURY procedure (*p* = 0.014) to discriminate PCa from BPH samples (Table 4). However, when PSA was introduced in the miRNA-based model, it resulted in higher predictive accuracy than PSA as biomarker as previously described [8], both to discriminate PCa from nonmalignant individuals (miR-142-3p + miR-142-5p + PSA model: AUC > 0.85, *p* < 0.009) and to discriminate benign from malignant tumours (miR-142-3p + miR-142-5p + miR-223-3p + PSA model: AUC > 0.933, *p* < 0.009) for the three sEV extraction methods (Table 4). Interestingly, a better use for diagnosis of these combined miRNA-PSA models than PSA as biomarker was also obtained, resulting in a higher sensitivity and specificity to discriminate not only PCa from healthy individuals, but, more clinically relevant, to discriminate malignant from benign tumours (Table 4) for the three sEV extraction methods.

## 3. Discussion

Over the last decade, many studies have identified semen as an ideal “liquid biopsy” that could be used for biomarker identification to improve the diagnosis and prognosis of male reproductive system disorders [9]. Specifically, semen exosomes could be a useful source of miRNA biomarkers for the diagnosis of urogenital diseases such as male infertility [7] and prostate cancer [8]. However, the analysis of miRNAs from this semen fraction can be technically challenging because exosome isolation is currently based on differential ultracentrifugation, which may not be available in the clinical laboratories.

In fact, although there is increasing interest in exosome research amongst the scientific community, few exosomal biomarkers have been implemented in clinical practice, due to the fact that exosome-sEV isolation and characterization in some biofluids remains a challenge for scientists [10]. Ultracentrifugation is considered to be the quintessential technique for isolating exosomes/EVs from biological fluids [11,12]. It is estimated that this method represents 56% of all exosome isolation techniques used by researchers, as it is easy to use and does not require a long pre-treatment of samples or much technical experience [13]. However, it requires the right equipment in the laboratory—this is usually expensive—and the protocol is quite time-consuming. Recently, several commercial kits have appeared on the market for the isolation and study of exosomes based on precipitation reagents and/or on application of columns. Compared to ultracentrifugation, these methods consume less time, are more compatible with a limited volume of biological samples and do not require special equipment [14]. However, their protocols focus on urine samples, blood serum/plasma or conditioned media from cells in culture, so that in order to use them with other fluids such as semen samples, they will need to be optimized. Additionally, there is no previous evidence of the effect of these semen exosome-EV isolating method on subsequent molecular analysis, thus it is necessary to assess their sEV purifying effectiveness and their impact on the downstream analysis of the molecules they contain such as miRNAs against the standard use of ultracentrifugation. To our knowledge, our study is the first research analysis addressing different exosome-EV isolation methods from semen for miRNA profiling.

In the present study, we have been able to verify first of all, that commercial kits designed to isolate exosomes and other EVs from serum, plasma or urine are generally applicable to SP although EV recovery rates or isolation efficiency vary between them. Additionally, our results provide evidence that there are variations not only in EV purifying efficiency but also in EV-RNA quantity and quality between the different exosome-EV isolation methods. *ExoGAG* and *miRCURY Cell/Urine/CSF* kits proved to be the most efficient methods for use in semen samples. They provide nanoparticle diameter results that correspond to the established range for exosomes, and also the variations introduced in the centrifugation step (*miRCURY Cell/Urine/CSF* 1500× *g* and *ExoGAG* 3500× *g* kit) allowed us to obtain good results when compared with the *UC* method, both in terms of nanoparticle concentration and the quality of the RNA the vesicles contain. Among these two exosome-EV isolation methods, *miRCURY Cell/Urine/CSF 1500× g* showed the highest particle yield and recovery, but a lower RNA purity, although it was still suitable for a subsequent miRNA quantitative analysis. When abundant miRNAs in semen exosomes were amplified in control samples, we obtained similar raw Cp values for these two methods of EV extraction to those obtained with *UC*. Accordingly, these two protocols, together with *UC*, were selected to determine their effects on the analysis of the level of miRNAs contained in the vesicles obtained from samples from patients with pathological conditions.

Additionally, in the next phase of the study we observed that, even when similarly efficient EV isolating methods are used, the expression profile of the miRNAs in the different groups of the PCa study varied depending on the method of exosome-EV extraction applied. Male samples can be discriminated as having PCa or not by using the expression values of some sEV-miRNAs as biomarkers; however, our results provide evidence indicating that miRNAs are differentially expressed in PCa and can vary depending on the extraction method. Interestingly, the EV isolation method has previously been described as introducing bias in the EV miRNA profile from cell culture medium and serum [15,16,17], which corroborates with our results in semen. There are several reasons that could explain the impact of the EV extraction method on the level of the exosomal miRNAs quantified. First, the *ExoGAG* and *miRCURY Cell/Urine/CSF* kits, as well as *UC*, can be used to isolate the extracellular vesicles below 200 nm for the analysis of the expression patterns of miRNAs they contained, but the small differences in the diameter of isolated nanovesicles, differences in CD63 and CD81 protein levels on sEVs, and differences in the expression patterns of miRNAs observed between the three methods (*UC*, *ExoGAG* and *miRCURY Cell/Urine/CSF*) suggest that the proportion of exosomes present in the nanovesicle extract varies among the different isolation methods applied. What is more, the observed over-representation of specific miRNA from vesicles obtained by a particular isolation method (such as miR-663b for sEVs isolated with *ExoGAG* method) suggest some miRNAs are likely to be associated to specific vesicle sets enriched by this particular method of isolation. In general, both healthy and pathological cells release vesicles of varying sizes [18]. In our study, thanks to the filtration of the SP that we carried out prior to sEV extraction, we removed vesicles larger than 200 nm (apoptotic bodies and bigger microvesicles) as previously described [6,18]. However, the extract enriched with particles smaller than 200 nm can include sEV subpopulations in different proportions, both exosomes and the fraction of microvesicles that exhibit a diameter similar to exosomes, which are hardly indistinguishable by current techniques such as Western or flow cytometry, since both microvesicles and exosomes have similar densities and share the same membrane proteins/markers. It is worth noting that exosomes and microvesicles are formed by different biogenesis mechanisms, which is likely to affect their cargo [2], thus a varying composition of purified vesicle preparation will affect the final sEV-miRNA quantification. In fact, the term “exosome”, extensively used in the literature for those EVs of small size recovered by ultracentrifugation and other methods of isolation, has been suggested to be redefined with a more appropriate term such as “sEV” as the heterogeneity of EVs isolated from cell culture media and biofluids has become obvious [19]. Our data suggests that the method used for EV isolation from semen determine the composition of the nanovesicle preparation, introducing a bias for subsequent miRNA analysis.

Additionally, the EV isolation technique of reference, *UC*, has been described to induce aggregation of EVs [20], which may have consequences for the structural integrity of EVs and consequently on the final miRNA analysis.

All these data support that, the detected fluctuations of miRNA levels between methods are probably due to the differences in the composition of sEV subtypes isolated with *UC*, *miRCURY* and *ExoGAG* methods.

Interestingly, previously described semen exosome miRNA signatures that combined with blood PSA concentration as molecular biomarkers with the potential to improve PCa diagnosis efficiency of PSA [8] were assessed for the three sEV isolation methods. The impact of EV extraction methods was reduced in PSA-miRNA combined models that conserve the statistical power as molecular classifier to distinguish benign from malignant tumours with any of the three EV isolation techniques and thus, have the potential to reduce unnecessary biopsies in the PSA “grey zone” (PSA between 4–10 ng/mL). Therefore, the miRNA-PSA combined models can be useful for any of the three procedures of EV isolation: *UC*, *ExoGAG 3500× g* and *miRCURY Cell/Urine/CSF 1500× g* methods, improving the diagnostic performance of blood PSA for PCa.

One drawback of our study is that the number of analysed samples is relatively low. An increase in the number of samples would provide more statistical strength by reducing the effect of variability; this is important in the case of those miRNAs that shared the same expression trend in the study groups but showed statistically different values when using alternative EV isolation methods. Therefore, to identify relevant sEV biomarkers detected after using either isolation method, we propose broadening the search for more miRNAs contained in the exosomes/sEVs of the SP and studying them in a larger number of samples using different extraction methods in parallel.

With this study, a research framework for the extraction of exosomes/sEVs from the SP has been set up, which was nonexistent until now. It has shown that different methods of isolation of extracellular vesicles produce variations not only in the concentration, but also in the size of EVs, probably as a result of isolating different proportion of sEV subpopulations. This fact determines that the EV isolation method influences the sEV profile and the consequent result of the downstream analysis of the molecules they contain. Thus, our study provides evidence that the isolation method of EVs has a great impact on the analysis of the EV miRNAs, which could lead to misleading results and conclusions, with important consequences for their use as clinical biomarkers. This impact could be reduced in clinically useful miRNA-PSA combined models, which improve the accuracy of PSA in the management of early PCa diagnosis.

In summary, the identification of the optimal technique to isolate the exosomes contained in SP is essential for the identification of PCa biomarkers. Even more so, it is necessary to assess their future applicability in clinical laboratories, where the available devices and technology must be taken into account, not only for diagnosis but also for exosome-based therapeutic purposes. Our study shows an impact of the isolation method not only on the number of the vesicles isolated but also in the type, content and integrity of these vesicles. Accordingly, the results of the analysis of miRNA expression contained in sEVs, either as single or combined biomarkers, cannot be directly extrapolated between different exosome-EV isolation methods for clinical application until clear markers for delineation between sEV subtypes are established.

## 4. Materials and Methods

### 4.1. Subjects of Study

Semen specimens were collected from healthy individuals without tumours (control group 1: HCt; n = 12) consulting for vasectomy or infertility at the Andrology Service of Fundació Puigvert and individuals consulting for diagnosis of PCa who underwent routine prostate screening including PSA testing and DRE (digital rectal examination) at the Urology Service of the Bellvitge Hospital; only patients that presented moderate PSA levels (4–18 ng/mL) and consented to undergo prostate biopsy were selected. The patient group (PCa) comprised 9 men with biopsy-proven PCa including those who were previously successfully vasectomised (PCa-V, n = 2) and non-vasectomised individuals (PCa-noV, n = 7). Additionally, samples from 5 non-vasectomised individuals with benign prostatic hyperplasia (BPH) or prostate enlargement (control group 2: BPH) who presented elevated PSA levels (>4 ng/mL) but no detectable cancer on biopsy (Table 1), were collected. The study was approved by the Ethical Review Board of both institutions, Fundació Puigvert and Bellvitge Hospital (Feb 2015, 2018). The participants signed an informed consent form. All methods were performed in accordance with the relevant guidelines and regulations.

Samples were analysed in two phases: the pre-study, to optimize and select the proper exosome-EV isolation methods for semen, and the study, to analyse the miRNA expression levels in the different groups of study and compare the results among the suitable EV isolation methods for semen. The pre-study included 7 HCt samples, whereas the study included 5 HCt, 5 BPH and 9 PCa samples (Table 1). In order to avoid miRNA expression differences due to inter-individual variations, the same semen samples were used for the different isolation methods within the pre-study as well as within the study phase.

### 4.2. Sample Collection and Processing

Semen samples were obtained by masturbation after 3–5 days of sexual abstinence. They were allowed to liquefy for 30 min at 37 °C. Samples were centrifuged twice (1600× *g* for 10 min, then 16,000× *g* for 10 min) at 4 °C to obtain seminal plasma (supernatant) as previously described [7,8,21]. Seminal plasma (SP) was carefully collected and immediately stored at −80 °C until use.

### 4.3. Exosome/sEV Isolation (Figure 1)

SP (200 µL) was first passed through a 0.22 μm filter to enrich for sEVs [6]. The resulting filtrate was processed by either ultracentrifugation, three commercial exosome-EV isolation reagents [*miRCURY^®^ Exosome Cell/Urine/CSF kit* (*Qiagen*, NV; Germany), *miRCURY^®^ Exosome Serum/Plasma kit* (*Qiagen*, NV; Germany), *ExoQuick^®^ ULTRA EV Isolation kit for Serum and Plasma* (*System Biosciences, Palo Alto, CA, USA*)] or a non-commercial exosome-EV isolation reagent [*ExoGAG* (NasasBiotech, Santiago de Compostela, Spain)] previously tested for isolating plasma EV from endometrial cancer patients [22]. They all were precipitation-based methods, although *ExoQuick^®^ ULTRA EV Isolation kit for Serum and Plasma* can also include a final solid extraction phase (precipitation + column-based method). Different experimental conditions were tested for each isolation reagent (Figure 1). *Ultracentrifugation (UC)*.

The filtered SP fluid plus 9 mL of PBS was ultra-centrifuged at 100,000× *g* in a SW40 rotor for 2 h at 4 °C to sediment the sEVs, which mainly contain exosomes, as described elsewhere [17]. The pellet was resuspended in 100 µL PBS and frozen at −80 °C.

- *miRCURY ^®^ Exosome Cell/Urine/CSF kit (miRCURY Cell/Urine/CSF)*

The exosomal fraction from SP filtered fluid was isolated according to the manufacturer’s recommendations. Briefly, filtered fluid was supplemented with PBS till 1mL and subsequently, 400 µL of Precipitation buffer B was added. Samples were incubated at 4 °C for 1 h. The mixture was centrifuged at either 10,000× *g* (manufacturer’s protocol) or 1500× *g* for 30 min at room temperature. Pellet fraction was resuspended in 100 µL of Resuspension buffer and frozen at −80 °C.

- *miRCURY ^®^ Exosome Serum/Plasma kit (miRCURY S/P)*

Both protocols (for serum and for plasma) were independently followed. SP filtered fluid was supplemented with PBS till 0.5 mL. Then the corresponding amounts of reagents were added according to the manufacturer’s instructions. Samples were incubated at 4 °C for 1 h and centrifuged at 1500× *g* 30 min (protocol serum) or 500× *g* 5 min (protocol plasma) at room temperature. Pellet fraction was resuspended in 270 µL of Resuspension buffer and frozen at −80 °C.

- *ExoQuick^®^ ULTRA EV Isolation kit for Serum and Plasma (ExoQuick Ultra_S/P)*

The corresponding amounts of *ExoQuick^TM^ EV isolation reagent* was added to 0.2 mL of filtered fluid according to the manufacturer’s instructions. Samples were incubated at 4 °C for 30 min or overnight. The seminal plasma/*ExoQuick* mixture was centrifuged at 1500× *g* [23] or 3000× *g* for 10 min at 4 °C and the pellet resuspended in 100 µL PBS (protocol B: precipitation) and frozen at −80 °C. For several samples, a column step was additionally followed (protocol A: precipitation + column) for comparison.

- *ExoGAG*

A suitable volume of precipitation reagent A of *ExoGAG* kit was added to 0.2 mL of filtered seminal plasma (reagent A/sample ratio 2:1) [22]. The mixture was incubated for 5 min at 4 °C and subsequently centrifuged at either 1500× *g* or 3500× *g* for 30 min at 4 °C. The pellet was resuspended in 100 µL PBS and frozen at −80 °C.

### 4.4. Characterization of EVs

Vesicles were characterized in terms of physical properties (size, concentration) by Nanoparticle tracking analysis (NTA) using the NanoSight NS300 (Malvern Instruments).

An additional flow cytometry analysis (BD FACSAria^TM^ IIu, BDbiosciences, SanJosé, CA, USA) was performed and tetraspanins CD63, and CD81 protein levels were analysed as the most usually employed markers for EV characterization [19,24]; their presence demonstrates the lipid-bilayer structure specific of EVs. Semen EVs isolated by *UC*, *ExoGAG* or *miRCURY cell/urine/CSF* were incubated for 1 h with antiCD81 (1:50, sc7637, Santa Cruz Biotechnology, Santa Cruz, CA, USA), antiCD63 (1:50, sc5275, Santa Cruz Biotechnology, Santa Cruz, CA, USA) and antiIgG (1:50, 400120, BioLegend) as negative control, followed by anti-mouse Alexa488 (1:1000, ab150113, Abcam, Cambridge, UK) as the secondary antibody as described [22]. Quantification of fluorescence was calculated as the fold-change of fluorescence intensity median with respect to IgG (immunoglobulin G) negative control. Positive signals were considered as those with a fold-change fluorescence intensity of two or higher than two.

### 4.5. Small RNA-Containing Total RNA Isolation from Semen Exosomes/sEVs

With the purpose of degrading the residual RNA outside the vesicles, the vesicle suspension was treated with RNAse A (Qiagen; NV, Germany) (100 μg/mL final reaction concentration; 15 min at 37 °C). Total RNA was obtained from exosomes/sEVs using the *miRNeasy Micro Kit* (Qiagen; NV, Germany). RNA concentration was calculated by using the QUBIT fluorometer and the Quant-iT RNA Assay kit (Invitrogen; CA, USA). RNA quality was determined by evaluating the OD 260/280 nm ratio when using a Nanodrop UV-Vis spectrophotometer (Thermo Fisher Scientific; MA, USA).

### 4.6. Exosomal miRNA Quantitative Real-Time PCR (qPCR)

First-stranded cDNA specific for miRNA was obtained by reverse transcription (RT) of 50 ng of exosomal RNA in 10 µL, using the *miRCURY^®^ LNA^®^ RT* Kit (Qiagen; NV; Germany). For qPCR analysis, cDNA was diluted (12×) and assayed in 10 µl PCR reactions containing *miRCURY LNA SYBR^®^ Green PCR* Kit (Qiagen; NV; Germany). Duplicate amplification reactions of 16 individual miRNA assays (LNA™-enhanced miRNA qPCR primers; Supplementary Materials Table S2), including miRNAs previously described as altered in PCa exosome semen samples [8], were carried out on a Lightcycler^®^ 96 Instrument (Roche; Basilea, Switzerland). Target miRNA expression in semen sEV samples was calculated relative to the mean expression value of the most stable assays as normalizers, to correct for potential overall differences between the samples. Normalizers (miR-576-5p and miR-181c-5p) were selected because they presented the lowest coefficient of variation (CV < 0.030) of Cp values among the samples in the study (Table 3), as well as having no statistical differences in absolute expression levels between groups from all the extraction methods used (Table 3). The relative quantification (RQ) miRNA expression values were calculated using the 2^dCp^ strategy.

### 4.7. Statistical Analysis

Statistical analysis was carried out with nonparametric tests such as the Kruskal–Wallis and Mann–Whitney tests to evaluate differences among the groups of study in all exosome isolation methods.

Receiver operating characteristic (ROC) curve analysis of the RQ values was used to distinguish the samples showing malignancy of prostate tumour. Accuracy was measured as the area under the ROC curve (AUC) [25]. The threshold value was determined by Youden’s index, calculated as sensitivity plus specificity-1 [26]. A multivariate binary logistic regression analysis was used to assess whether previously described miRNA combined models [8] were useful as PCa biomarkers from sEVs obtained from the different EV isolation methods. An enter method was used to include in the equation all the variables of each nested model. The binary logistic regression model provides the following estimation of the logit function:

Logit(p) = B0 + B1 × 1 + B2 × 2 + …

where p = P (presence of prostate cancer), Logit (p) = log(p/(1 − p)) = log(odds), B = logOR and Xn = the expression values of the miRNAs. Therefore, if we use this estimated model as a prediction model, with the standard classification cutoff of 0.5, we would classify individuals with a positive Logit function estimation as “positive for PCa” and individuals with negative Logit function estimation as “negative for PCa”.

Only *p*-values ≤ 0.05 were considered significant. All data analyses were performed using SPSS software, version 15 (SPSS Inc, Chicago, USA).

## Figures and Tables

**Table 1:** Clinical details of individuals included in this study.

	Pre-Study	Study		
Variable	HCt	HCt	BPH	PCa
Total, n	7	5	5	9
Age, mean ± SD	36.4 ± 6.2	42.2 ± 6.3	58.6 ± 4.3	57 ± 5.8
PSA pre-biopsy (n)				
≤10 (ng/mL)	-	-	5	7
>10 (ng/mL)	-	-	0	2
PSA pre-biopsy, mean ± SD (ng/mL)	-	-	5.5 ± 2.5	7.5 ± 3.3
Gleason score -biopsy (n)				
6 (3 + 3)	-	-	-	5
7 (3 + 4)	-	-	-	2
7 (4 + 3)	-	-	-	1
8 (4 + 4)	-	-	-	1
Clinical stage (n)				
cT1c	-	-	-	7
cT2a	-	-	-	-
cT2c	-	-	-	-
cT3a	-	-	-	2
Vasectomized (n)	-	5	0	2

HCt: healthy control group; BPH: benign prostate hyperplasia group; PCa: prostate cancer group.

**Table 2:** Comparison of the quality and quantity of exosomal RNA.

Extraction Method	[RNA] (ng/µL)	260/280	260/230
*Ultracentrifugation* (n = 5)	22.39 ± 7.79	1.78 ± 0.17	0.60 ± 0.40
*ExoGAG 1500× g* (n = 2)	8.17 ± 2.55	1.76 ± 0.05	0.13 ± 0.05
*ExoGAG 3500× g* (n = 4)	12.29 ± 8.6	1.79 ± 0.08	0.47 ± 0.40
*ExoQuick ULTRA A 3000× g* (n = 2)	4.27 ± 0.94	1.53 ± 0.04	0.39 ± 0.18
*ExoQuick ULTRA B 3000× g* (n = 2)	4.25 ± 0.93	1.48 ± 0.09	0.38 ± 0.08
*ExoQuick ULTRA A 1500× g* (n = 1)	<2	1.52	0.51
*ExoQuick ULTRA B 1500× g* (n = 1)	7.73	1.51	0.72
*miRCURY cell/urine/CSF 10,000× g* (n = 2)	3.65 ± 3.04	1.17 ± 0.01	0.10 ± 0.01
*miRCURY cell/urine/CSF 1500× g* (n = 4)	31.03 ± 2.94	1.63 ± 0.11	0.40 ± 0.26
*miRCURY Serum* (n = 2)	4.86 ± 3.97	1.69 ± 0.09	0.54 ± 0.36

A260/280 and A260/230 ratios of a nanodrop spectrometer for RNA samples purified from exosomal extracts. RNA concentration was calculated with a Qubit device. Data is expressed as mean ± SD.

**Table 3:** Summary of miRNA expression data in semen vesicles obtained with the three most efficient EV isolation methods.

A.									
	Average Cq *ULTRACENTRIFUGATION*				*p*-value	miRNA expression			*p*-value
Gene Name	HCt	BPH	PCa	CV	(HCt-BPH-PCa)	HCt	BPH	PCa	HCt-PCa
let7i-3p	33.39	33.01	36.14	0.076	0.049	1	1.360	0.395	0.083
miR 106b-5p	24.15	23.00	24.64	0.075	>0.10	1	1.905	1.083	>0.10
miR 125a-5p	21.94	21.03	21.85	0.032	>0.10	1	1.417	1.270	0.060
miR 130a-3p	28.41	26.96	28.09	0.042	>0.10	1	1.986	1.466	>0.10
miR 142-3p	29.51	27.10	28.19	0.056	0.059	1	5.309	3.066	**0.042**
miR 142-5p	31.30	29.93	31.43	0.060	>0.10	1	2.291	1.443	>0.10
miR 181c-5p	30.10	30.17	30.52	0.027	>0.10	1	0.694	0.845	>0.10
miR 196b-3p	29.03	29.63	30.14	0.024	**0.014**	1	0.498	0.562	**0.004**
miR 223-3p	29.54	27.02	29.69	0.105	>0.10	1	7.742	2.627	>0.10
miR 30c-5p	22.31	20.20	21.57	0.059	**0.038**	1	3.904	1.892	**0.042**
miR 30e-3p	25.45	23.22	24.75	0.047	**0.007**	1	4.139	1.746	0.060
miR 34a-3p	30.11	28.24	29.38	0.041	0.050	1	3.452	2.080	**0.019**
miR 34a-5p	25.46	24.40	25.52	0.037	**0.027**	1	1.682	1.172	>0.10
miR 576-5p	30.66	29.78	30.65	0.026	0.055	1	1.348	1.134	>0.10
miR 663b	28.03	29.03	29.68	0.045	0.078	1	0.318	0.267	>0.10
miR 92a-3p	21.34	20.41	21.34	0.037	**0.034**	1	1.657	1.169	>0.10
*miR (576+181)*				*0.024*					
**B.**									
	**Average Cq *miRCURY cell/urine/CSF***				***p*** **-value**	**miRNA expression**			***p*** **-value**
**Gene Name**	**HCt**	**BPH**	**PCa**	**CV**	**(HCt-BPH-PCa)**	**HCt**	**BPH**	**PCa**	**HCt-PCa**
let7i-3p	31.43	30.75	32.27	0.110	>0.10	1	2.17	1.60	>0.10
miR 106b-5p	22.81	22.86	23.32	0.050	>0.10	1	0.82	0.79	>0.10
miR 125a-5p	21.30	21.32	21.74	0.031	>0.10	1	0.80	0.79	>0.10
miR 130a-3p	26.78	25.38	26.18	0.041	0.076	1	2.66	1.62	0.060
miR 142-3p	28.34	26.69	27.19	0.069	>0.10	1	3.26	2.44	>0.10
miR 142-5p	30.16	28.45	29.02	0.058	>0.10	1	3.52	2.41	>0.10
miR 181c-5p	30.02	29.80	30.16	0.035	>0.10	1	0.97	0.96	>0.10
miR 196b-3p	28.76	28.87	28.86	0.027	>0.10	1	0.77	1.04	>0.10
miR 223-3p	28.81	27.21	28.52	0.072	>0.10	1	2.01	0.90	>0.10
miR 30c-5p	21.12	20.85	21.14	0.042	>0.10	1	0.98	1.05	>0.10
miR 30e-3p	24.75	24.34	24.82	0.037	>0.10	1	1.04	1.02	>0.10
miR 34a-3p	28.50	28.13	28.30	0.031	>0.10	1	1.00	1.12	>0.10
miR 34a-5p	24.84	24.63	25.00	0.036	>0.10	1	0.97	0.91	>0.10
miR 576-5p	29.91	29.60	29.90	0.029	>0.10	1	1.03	1.05	>0.10
miR 663b	28.33	26.97	27.19	0.043	0.077	1	2.15	4.53	>0.10
miR 92a-3p	20.53	20.48	20.77	0.031	>0.10	1	0.84	0.98	>0.10
*miR (576+181)*				*0.029*					
**C.**									
	**Average Cq *EXOGAG***				***p*** **-value**	**miRNA expression**			***p*** **-value**
**Gene Name**	**HCt**	**BPH**	**PCa**	**CV**	**(HCt-BPH-PCa)**	**HCt**	**BPH**	**PCa**	**HCt-PCa**
let7i-3p	35.24	34.04	34.69	0.026	0.074	1	2.01	1.90	>0.10
miR 106b-5p	25.25	25.09	25.34	0.043	>0.10	1	1.04	1.17	>0.10
miR 125a-5p	21.86	22.18	22.73	0.029	0.054	1	0.71	0.72	0.060
miR 130a-3p	28.74	27.42	28.25	0.035	>0.10	1	2.03	1.69	0.083
miR 142-3p	31.23	28.99	29.88	0.057	**0.038**	1	5.05	3.34	**0.042**
miR 142-5p	32.27	30.78	31.72	0.043	>0.10	1	2.59	1.93	>0.10
miR 181c-5p	31.25	30.77	31.17	0.022	>0.10	1	1.27	1.37	>0.10
miR 196b-3p	29.19	29.87	29.37	0.031	>0.10	1	0.55	1.05	>0.10
miR 223-3p	29.08	27.94	28.41	0.053	>0.10	1	3.31	1.83	>0.10
miR 30c-5p	21.89	21.95	22.41	0.038	>0.10	1	0.87	0.90	>0.10
miR 30e-3p	26.58	26.66	27.04	0.039	>0.10	1	0.93	0.96	>0.10
miR 34a-3p	29.66	29.23	29.83	0.029	>0.10	1	1.15	1.12	>0.10
miR 34a-5p	26.91	26.50	27.17	0.044	>0.10	1	1.13	1.11	>0.10
miR 576-5p	30.18	30.33	30.96	0.028	>0.10	1	0.81	0.73	>0.10
miR 663b	24.53	24.46	24.55	0.043	>0.10	1	0.65	1.25	>0.10
miR 92a-3p	21.63	22.40	22.79	0.041	**0.045**	1	0.57	0.61	**0.042**
*miR (576+181)*				*0.022*					

**Table 4:** Diagnostic efficiency of multivariate logistic analysis including previously described miRNA-based models [8] in the different extraction strategies of the study.

**PCa vs. non-PCa (HCt+BPH)**	[*PSA*] model	[*miR-142-3p+miR-142-5p*] model			[*miR-142-3p+miR-142-5p+PSA*] model		
		*UC*	*miRCURY Cell_U_CSF*	*ExoGAG*	*UC*	*miRCURY Cell_U_CSF*	*ExoGAG*
AUC	0.844	0.556	0.644	0.667	0.856	0.867	0.922
95% CI	0.668–1.021	0.283–0.828	0.385–0.904	0.409–0.924	0.661–1.050	0.681–1.053	0.804–1.040
*p*-value	**0.011**	0.683	0.288	0.221	**0.009**	**0.007**	**0.002**
Sensitivity (%)	66.7	22.2	22.2	11.1	77.8	77.8	66.7
Specificity (%)	80.0	90.0	90.0	90.0	90.0	90.0	90.0
Positive predictive value	75.0	66.7	66.7	50.0	87.5	87.5	85.7
Negative predictive value	72.7	56.3	56.3	52.9	81.8	81.8	75.0
**PCa vs. BPH**	[*PSA*] model	[*miR-142-3p+miR-142-5p+miR-223-3p*] model			[*miR-142-3p+miR-142-5p+miR-223-3p+PSA*] model		
		*UC*	*miRCURY Cell_U_CSF*	*ExoGAG*	*UC*	*miRCURY Cell_U_CSF*	*ExoGAG*
AUC	0.689	0.644	0.911	0.467	0.933	1	1
95% CI	0.398–0.979	0.309–0.980	0.754–1.069	0.066–0.867	0.790–1.076	1.000–1.000	1.000–1.000
*p*-value	0.257	0.386	**0.014**	0.841	**0.009**	**0.003**	**0.003**
Sensitivity	100.0	100.0	88.9	100.0	100.0	100.0	100.0
Specificity	20.0	40.0	80.0	40.0	80.0	100.0	100.0
Positive predictive value	100.0	75.0	88.9	75.0	90.0	100.0	100.0
Negative predictive value	69.2	100.0	80.0	100.0	100.0	100.0	100.0

## Supplementary Materials

Supplementary Materials can be found at https://www.mdpi.com/1422-0067/21/17/5949/s1. Table S1. Nanoparticle tracking analysis (NTA) measurement of particle size of EV-extracts obtained with the different methods used; Table S2. List of the selected miRNAs and their sequences.

## Abbreviations

BPH	Benign prostate hyperplasia group
EV	Extracellular vesicle
HCt	Healthy control group
mRNA	Messenger RNA
miRNA	MicroRNA
NTA	Nanoparticle tracking analysis
PCa	Prostate cancer
PSA	Prostate specific antigen
RT-qPCR	Reverse transcription quantitative polymerase chain reaction
sEV	Small extracellular vesicle
SP	Seminal plasma
UC	Ultracentrifugation
