# Supplementary material for: Impact of Extracellular Vesicle Isolation Methods on Downstream miRNA Analysis in Semen: A Comparative Study
Source: Int J Mol Sci. 2020 Aug 19;21(17):5949. doi: 10.3390/ijms21175949 (PMC7504614; doi:10.3390/ijms21175949)
Supplement: Supplementary file 1 [file ijms-21-05949-s001.pdf]

Supplementary data

Supplementary Table S1. Nanoparticle tracking analysis (NTA) measurement of particle size of EV-extracts obtained with the different methods used

A)

	Cumulative percentage of particle distribution (%)		
Extraction method	105 (nm)	155 (nm)	255 (nm)
Ultracentrifugation (n=5)	21,2	72,5	91,3
Exo-GAG 1500xg (n=2)	28,3	82,2	95,7
Exo-GAG 3500xg (n=4)	25,3	83,7	94,0
ExoQuick ULTRA A 3000xg (n=2)	6,3	31,4	56,0
ExoQuick ULTRA A 1500xg (n=1)	12,7	48,1	73,5
ExoQuick ULTRA B 3000xg (n=2)	12,3	65,9	85,6
ExoQuick ULTRA B 1500xg (n=1)	10,7	62,0	90,6
miRCURY Cell/Urine/CSF 10000xg (n=2)	8,8	61,8	77,8
miRCURY Cell/Urine/CSF 1500xg (n=2)	19,6	70,9	88,1
miRCURY Serum (n=2)	14,2	69,9	85,9

B)

Extraction method	Size mean (nm)	Size mode (nm)	Size D10 (nm)	Size D50 (nm)	Size D90 (nm)
**Ultracentrifugation (n=5)**	147,26±4,44	119,10±1,96	53,12±3,39	91,62±1,66	123,22±3,96
**ExoGAG 1500x g (n=2)**	134,05±4,75	115,20±0,30	86,50±1,60	114,15±3,10	176,00±4,95
**ExoGAG 3500x g (n=4)**	135,23±2,52	117,45±1,83	45,95±4,06	87,53±0,95	113,93±1,31
**ExoQuick ULTRA A 3000x g (n=2)**	223,10±5,40	144,10±7,40	106,25±6,90	185,45±3,95	351,00±3,65
**ExoQuick ULTRA B 3000x g (n=2)**	158,85±7,05	138,65±14,75	97,60±7,00	134,90±0,90	218,60±6,40
**ExoQuick ULTRA A 1500x g (n=1)**	182,6	128	101,3	139,9	327,9
**ExoQuick ULTRA B 1500x g (n=1)**	153,6	119,1	100,6	130,1	200,9
**miRCURY cell/Urine/CSF 10000x g (n=2)**	184,65±6,95	122,85±0,25	101,05±4,95	133,70±1,45	337,00±3,60
**miRCURY cell/Urine/CSF 1500x g (n=2)**	152,40±3,20	114,30±1,60	63,55±0,85	92,45±0,15	124,70±3,20
**miRCURY Serum (n=2)**	157,55±1,05	123,25±5,35	66,75±2,75	95,70±1,90	128,10±0,40

Supplementary Table S2. List of the selected miRNAs and their sequences.

miRNA	5′- 3′ Sequence	No. nucleotides	miRNA PCa-associated Publication reference
let-7i-3p*	CUGCGCAAGCUACUGCCUUGCU	22	
miR-106b-5p	UAAAGUGCUGACAGUGCAGAU	21	
miR-125a-5p	UCCCUGAGACCCUUUAACCUGUGA	24	Fu et al.,2015. Oncotarget
miR-130a-3p	CAGUGCAAUGUUAAAAGGGCAU	22	Zhang et al., 2019. Eur Rev. Med Pharmacol Sci
miR-142-3p*	UGUAGUGUUUCCUACUUUAUGGA	23	Barceló et al., 2019 Sci Rep; Tan et al., 2020 J Cancer
miR-142-5p*	CAUAAAGUAGAAAGCACUACU	21	Barceló et al., 2019 Sci Rep
miR-181c-5p	AACAUUCAACCUGUCGGUGAGU	22	
miR-196b-3p	UCGACAGCACGACACUGCCUUC	22	Jeong et al., 2017. Mol Cell
miR-223-3p	UGUCAGUUUGUCAAAUACCCCA	22	Barceló et al., 2019 Sci Rep; Feng et al., 2018 Gene; Wei et al., 2014 Sci Rep
miR-30c-5p	UGUAAACAUCCUACACUCUCAGC	23	Kumar et al., 2016 Oncotarget
miR-30e-3p	CUUUCAGUCGGAUGUUUACAGC	22	
miR-34a-3p	CAAUCAGCAAGUAUACUGCCCU	22	
miR-34a-5p	UGGCAGUGUCUUAGCUGGUUGU	22	Ma et a., 2019 Onco Targets Ther.
miR-576-5p	AUUCUAAUUUCUCCACGUCUUU	22	
miR-663b*	GGUGGCCCGGCCGUGCCUGAGG	22	
miR-92a-3p	UAUUGCACUUGUCCCGGCCUGU	22	Huo et al., 2020 Cancer Cell Int
